# Serum Cytokine Responses over the Entire Clinical-Immunological Spectrum of Human *Leishmania (L.) infantum chagasi* Infection

**DOI:** 10.1155/2016/6937980

**Published:** 2016-03-08

**Authors:** Patrícia Karla Ramos, Karina Inácio Carvalho, Daniela Santoro Rosa, Ana Paula Rodrigues, Luciana Vieira Lima, Marliane Batista Campos, Claudia Maria C. Gomes, Márcia Dalastra Laurenti, Carlos Eduardo Corbett, Fernando Tobias Silveira

**Affiliations:** ^1^Parasitology Department, Evandro Chagas Institute, Surveillance Secretary of Health, Ministry of Health, Ananindeua, PA, Brazil; ^2^Albert Einstein Israelite Hospital, São Paulo, SP, Brazil; ^3^Division of Immunology, Federal University of São Paulo, São Paulo, SP, Brazil; ^4^Pathology Department, Medical School of São Paulo University, São Paulo, SP, Brazil; ^5^Tropical Medicine Nucleus, Federal University of Pará, Belém, PA, Brazil

## Abstract

The clinical-immunological spectrum of human* Leishmania (L.) infantum chagasi* infection in Amazonian Brazil was recently reviewed based on clinical, DTH, and IFAT (IgG) evaluations that identified five profiles: three asymptomatic (asymptomatic infection, AI; subclinical resistant infection, SRI; and indeterminate initial infection, III) and two symptomatic (symptomatic infection, SI; American visceral leishmaniasis, AVL; and subclinical oligosymptomatic infection, SOI). TNF-*α*, IL-4, IL-6, and IL-10 serum cytokines were analyzed using multiplexed Cytometric Bead Array in 161 samples from endemic areas in the Brazilian Amazon: SI [AVL] (21 cases), III (49), SRI (19), SOI (12), AI (36), and a control group [CG] (24). The highest IL-6 serum levels were observed in the SI profile (AVL); higher IL-10 serum levels were observed in SI than in SOI or CG and in AI and III than in SOI; higher TNF-*α* serum levels were seen in SI than in CG. Positive correlations were found between IL-6 and IL-10 serum levels in the SI and III profiles and between IL-6 and TNF-*α* and between IL-4 and TNF-*α* in the III profile. These results provide strong evidence for associating IL-6 and IL-10 with the immunopathogenesis of AVL and help clarify the role of these cytokines in the infection spectrum.

## 1. Introduction

Although American visceral leishmaniasis (AVL) is considered the major clinical feature of* Leishmania (L.) infantum chagasi* and human immune response, there is no doubt that additional clinical-immunological features resulting from this interaction could also contribute to better understanding the entire immune response to infection. New findings concerning human* L. (L.) i. chagasi* infection in Pará State, Amazonian Brazil, based on the combined use of the semiquantitative delayed-type hypersensitivity (DTH) and indirect fluorescent antibody tests (IFAT-IgG) have confirmed their ability to diagnose either asymptomatic or symptomatic infections in endemic areas [1]. The high specificity of this diagnostic approach using species-specific* L. (L.) i. chagasi* antigens, promastigotes for DTH, and amastigotes for IFAT (IgG), associated with the clinical status of the infected individuals, has allowed the identification of a wide infection spectrum composed of five clinical-immunological profiles: three asymptomatic, (1) asymptomatic infection (AI) [DTH^+/++++^, IFAT^−^], (2) subclinical resistant infection (SRI) [DTH^+/++++^, IFAT^+/++^], and (3) indeterminate initial infection (III) [DTH^−^, IFAT^+/++^] and two symptomatic, (4) symptomatic infection (SI) [AVL] and (5) subclinical oligosymptomatic infection (SOI), both with the same immune profile [DTH^−^, IFAT^+++/++++^]. This diagnostic approach confirmed the three previously established profiles (AI, SI [AVL], and SOI) as well as two new ones (SRI and III) that represent new infection stages [2, 3].

**Figure 1:**
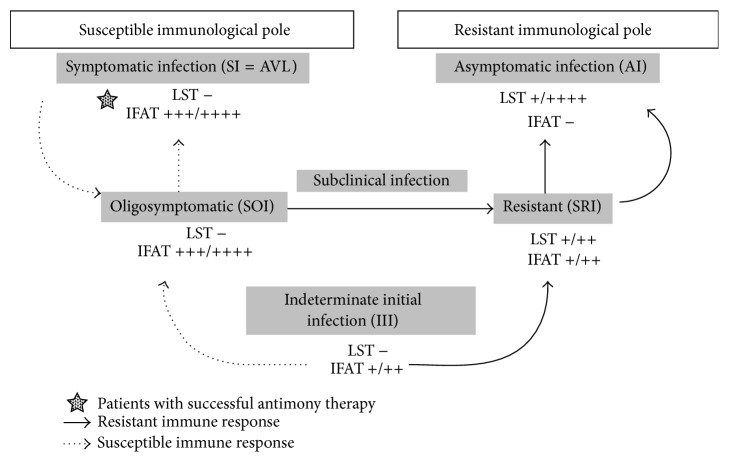
Dynamics of the clinical and immunological evolution of human* L. (L.) i. chagasi* infection in the Brazilian Amazon region. IFAT: indirect fluorescent antibody test (IgG). IFAT^++++^: 5120–10240 (IgG). IFAT^+++^: 1280–2560 (IgG). IFAT^++^: 320–640 (IgG). IFAT^+^: 80–160 (IgG). IFAT^−^: negative reaction. LST: Leishmanin skin test (= DTH). LST^++++^: exacerbate reaction (≥16 mm). LST^+++^: strong reaction (13–15 mm). LST^++^: moderate reaction (9–12 mm). LST^+^: weak reaction (5–8 mm). LST^−^: negative reaction. AI: asymptomatic infection. SI: symptomatic infection (= AVL). SOI: subclinical oligosymptomatic infection. SRI: subclinical resistant infection. III: indeterminate initial infection.

While the AI profile is characterized by a positive DTH reaction and the absence of an IgG-antibody response (below the IFAT-IgG dilution cut-off of 1 : 80) that are strongly linked to genetic resistance to infection [4], the SI profile (AVL) is associated with genetic susceptibility to infection, with strong inhibition of DTH and high expression of the IgG-antibody response. The SOI and SRI profiles represent borderline genetic expressions of susceptibility and resistance, respectively. The former is characterized by mild clinical signs of susceptibility (such as fever, asthenia, cutaneous pallor, and moderate enlargement of the spleen) but with spontaneous clinical resolution of the infection after one- to two-month period [5], while the latter is characterized by an asymptomatic profile representing an evolved stage towards the resistant AI profile. The asymptomatic III profile represents the earliest stage of infection, which is not well defined from an immune-genetic point of view but has the capacity to evolve into either a resistant AI profile or a susceptible SI (AVL) profile. Thus, depending on the genetic background of the infected individuals, the infection can evolve to a resistant AI profile or to a susceptible SI profile (AVL), although passing first through either the SRI or the SOI profile, respectively (Figure 1). We therefore undertook two prospective studies in the Brazilian Amazon focusing on the dynamics of the clinical and immunological evolution of infection and have estimated that 1–3% of the cases of the III profile will evolve to classical AVL [6, 7].

The innate immune response of the clinical-immunological profiles of human* L. (L.) i. chagasi* infection has not yet been exhaustively examined, as most studies have focused on the immunopathological disorders at the extreme pathogenic pole of this spectrum, the characteristic Th2-type cell response-derived SI profile (AVL). An immunopathogenic role of IL-10 (but not IL-4 or TGF-*β*) has been observed in* in vitro* T-cell suppression assays using cultured-stimulated peripheral blood mononuclear cells (PBMC) from cured AVL patients [8]; Caldas et al. [9] reported similar results for the plasma levels of IL-10 and IFN-*γ* from AVL patients, indicating that IL-10 can suppress the beneficial activity of IFN-*γ* activation of infected macrophages. We have also observed higher serum levels of IL-10 than IFN-*γ* in children with severe AVL, reinforcing the presumed immunopathogenic role of IL-10 [10]. IL-6 has recently been implicated, however, as one of most pathogenic inflammatory cytokines, being associated with infection severity and complications in children who later died from AVL in Piaui State in northeastern Brazil [11].

In terms of asymptomatic (AI, SRI, and III) and subclinical oligosymptomatic (SOI) infection profiles, a number of studies have confirmed the beneficial Th1 actions of IFN-*γ* and IL-12 cytokines in overcoming the Th2-inhibiting effect of IL-10 in cultured-stimulated PBMC from asymptomatic individuals with positive DTH reactions [12]. In two other experiments, following a short-term* L. (L.) i. chagasi*-soluble leishmania antigen/SLA stimulation of cultured whole peripheral blood, increased ratios of IFN-*γ* ^+^, TNF-*α* ^+^, IL-4^+^, and IL-10^+^ neutrophils and TNF-*α* ^+^ and IL-10^+^ monocytes were observed in asymptomatic individuals, suggesting a mixed pattern (Th1/Th2) of cytokine production due to innate immune responses in those individuals [13, 14]. The SOI profile demonstrated variable serum levels of IL-2 in 52.3% of the patients, IL-12 in 85.2%, IFN-*γ* in 48.1%, IL-10 in 88.9%, and TNF-*α* in 100%; IL-4 was not detected, confirming both Th1 and Th2 cytokine profiles in the subclinical oligosymptomatic form of infection [5]. The role of TNF-*α* within the infection spectrum is not yet fully understood; there is evidence for moderate plasma levels in asymptomatic individuals with positive DTH reactions, but high plasma levels in AVL patients showing negative DTH responses, suggesting a dubious role for TNF-*α* in infection outcomes [15–17].

Taking these observations into account, we present here our findings concerning TNF-*α*, IL-4, IL-6, and IL-10 serum cytokine responses over the entire clinical-immunological spectrum of human* L. (L.) i. chagasi*-infection, with emphasis on the immunopathogenic role of IL-6 and IL-10 which may aid in preventing or reducing the morbidity of severe AVL.

## 2. Materials and Methods

### 2.1. Study Area

The present study was carried out in the town of Santana do Cafezal, located on the banks of the Cafezal River, 7 km from the administrative center of the Municipality of Barcarena (01°30′S × 48°37′W), within the metropolitan region of Belém, the capital of Pará State, in the Brazilian Amazon. This area is dominated by plantations, with occasional patches of secondary forest. The climate and the environmental conditions of the study area were previously described in more detail [2].

### 2.2. Study Design and Clinical Samples

This cross-sectional study used flow cytometry to examine 161 serum samples from* L. (L.) i. chagasi*-infected and noninfected individuals (both genders, and ≥1 year old) from an endemic area of AVL in Pará State in the Brazilian Amazon. The individuals showed five clinical-immunological infection profiles: symptomatic infection (SI) [AVL] (21 cases), indeterminate initial infection (III) (49 cases), subclinical resistant infection (SRI) (19), subclinical oligosymptomatic infection (SOI) (12), and asymptomatic infection (AI) (36); 24 noninfected (DTH^−^/IFAT^−^) individuals (control group) were also examined. The criteria used for discriminating between these clinical-immunological infection profiles were described in previous studies [2, 5].

### 2.3. Cytokine Measurements by Flow Cytometry

Serum TNF-*α*, IL-4, IL-6, and IL-10 cytokines were detected simultaneously in a FACSCanto II flow cytometer using a cytokine Cytometric Bead Array (CBA) Human enhanced sensitivity flex set (BD PharMingen), according to the manufacturer's instructions. The assays were performed and analyzed by a single operator using FCAP array software. The detection range was 274–200,000 fg/mL for each cytokine.

### 2.4. Data Analysis

Bio-Estat 5.0 software was used to identify any statistically significant differences between the serum cytokine responses of the five clinical-immunological profiles. The mean cytokine responses of the five profiles and the negative control group were compared using the Mann-Whitney test and, when applicable, the 95% confidence interval was calculated for descriptive analyses of the data. The Spearman correlation test was used to analyze correlations between the cytokine responses of each clinical-immunological profile and the control group. The significance level was set at *P* ≤ 0.05.

## 3. Results

**Figure 2:**
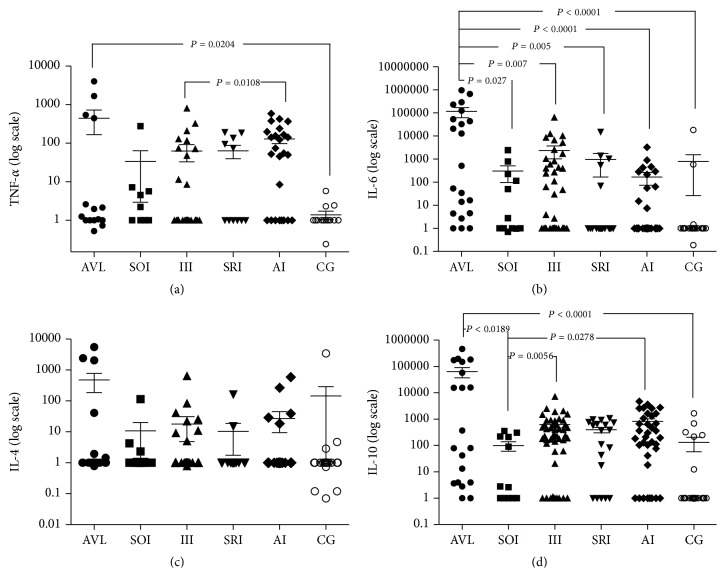
Flow cytometric assays showing the TNF-*α*, IL-4, IL-6, and IL-10 cytokine concentrations in 161 serum samples of* L. (L.) i. chagasi*-infected and noninfected individuals from an endemic area of AVL in the municipality of Barcarena, Pará State, in the Brazilian Amazon, representing the following five clinical-immunological profiles of infection: AVL = SI (21 cases), SOI (12), III (49), SRI (19), AI (36), and 24 noninfected (DTH^−^/IFAT^−^) individuals (CG). (a) TNF-*α*: tumor necrosis factor-alpha; (b) IL-6: interleukin-6; (c) IL-4: interleukin-4, and (d) IL-10: interleukin-10. AVL (American visceral leishmaniasis) = SI (symptomatic Infection profile); SOI (subclinical Oligosymptomatic Infection profile); III (indeterminate Initial Infection profile); SRI (subclinical Resistant Infection profile); AI (asymptomatic Infection profile); and CG (control Group).

The results of the present work were analyzed by comparing each cytokine response in terms of the different clinical-immunological infection profiles and the negative control group (Mann-Whitney test) and also by comparing each clinical-immunological infection profile and the negative control group with the different cytokine responses (Spearman correlation). Significantly higher IL-6 serum levels were found in the SI profile (AVL) in the first analysis as compared to the other profiles; significantly higher IL-10 serum levels were also observed in the SI profile (AVL) as compared to the SOI and the control group (CG) and in the AI and III profiles as compared to the SOI. Additionally, significantly higher TNF-*α* serum levels were detected in the SI profile (AVL) as compared to CG and in the AI profile as compared to the III. No significant differences in IL-4 serum levels were observed between the different clinical-immunological infection profiles (AI, SRI, III, SOI, and SI) and CG (Figure 2).

**Figure 3:**
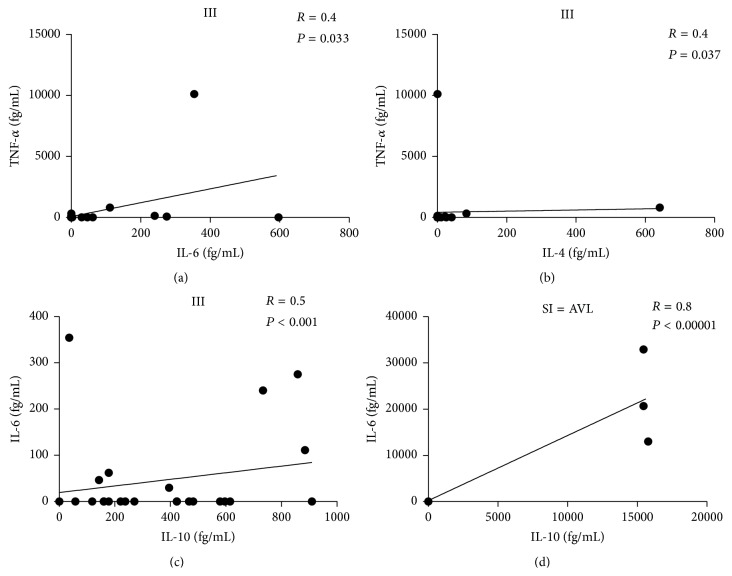
Positive correlations between the responses of IL-6 and IL-10 in the SI=AVL and III clinical-immunological profiles of human* L. (L.) i. chagasi* infection, as well as between IL-6 and TNF-*α* and IL-4 and TNF-*α* in the III profile from an endemic area of AVL in the municipality of Barcarena, Pará State, in the Brazilian Amazon. TNF-*α*: tumor necrosis factor-alpha; IL-6: interleukin-6; IL-4: interleukin-4; and IL-10: interleukin-10. III: indeterminate Initial Infection profile; SI = AVL: symptomatic Infection profile = American visceral leishmaniasis.

Positive correlations were observed in the second analysis between IL-6 and IL-10 serum levels in the SI [AVL] (*r*_*s*_ = 0.8 and *P* ≤ 0.05) and III (*r*_*s*_ = 0.5 and *P* ≤ 0.05) profiles, as well as between IL-6 and TNF-*α* and between IL-4 and TNF-*α* serum levels in the III (*r*_*s*_ = 0.4 and *P* ≤ 0.05) profile (Figure 3).

## 4. Discussion

This represents the first study carried out in Brazil that analyzed TNF-*α*, IL-4, IL-6, and IL-10 serum levels using flow cytometry along the wide clinical-immunological spectrum of human* L. (L.) i. chagasi*-infection composed of five different profiles (SI [AVL], SOI, III, SRI, and AI). Following infection, the antigenic stimuli of the parasite promotes first-line host defenses through the innate immune response which functions during the evolution of infection are sustained by the production of proinflammatory (TNF-*α*, IL-1*β*, IL-6, IL-8, IL-12, and IFN-*γ*) and anti-inflammatory (TGF-*β*, IL-4, and IL-10) cytokines. Here, we evaluated the role of some well-known prominent proinflammatory (TNF-*α* and IL-6) and anti-inflammatory (IL-4 and IL-10) cytokines within the entire clinical-immunological spectrum of human* L. (L.) i. chagasi*-infection to better understand their roles in the immunopathogenesis of infection.

It has been suggested that TNF-*α* is involved in acute inflammatory responses against Gram-negative bacterial infections, as well as other infectious agents [18, 19]. Its principal function appears to be the stimulation and recruitment of neutrophils and monocytes to infection sites and their activation to kill pathogenic microorganisms. TNF-*α* encodes genes that codify TNF-*α* and TNF-*β* and is associated with severe infectious diseases, including malaria, mucocutaneous leishmaniasis, visceral leishmaniasis, and tuberculosis; the latter two diseases are associated with high TNF-*α* serum/plasma levels [15–17, 20]. We demonstrated here significantly higher TNF-*α* serum levels in the SI (AVL) profile than in CG and in the AI profile as compared to III, demonstrating for the first time in Brazil that during early onset of infection with a negative DTH response (III profile) TNF-*α* serum levels are lower than those seen in resistant asymptomatic infections with positive DTH responses (AI profile) or in susceptible symptomatic infections with negative DTH responses (SI profile [AVL]). Based on these results, it seems reasonable to assume that, depending on the genetic profile for the DTH response of infected individual, the indeterminate initial infection (III profile with negative DTH) may evolve either with moderate TNF-*α* serum levels in resistant asymptomatic individuals (AI profile with positive DTH) or with elevated TNF-*α* serum levels in susceptible symptomatic patients (SI profile [AVL] with negative DTH), suggesting that the innate immune response-derived TNF-*α* protector effect is associated with other cell-mediated immunity mechanisms, such as delayed-type hypersensitivity (DTH). It should not be forgotten, however, that previous studies have shown that DTH and TNF-*α* expressions during the evolution of human* L. (L.) i. chagasi*-infection are strongly associated with the genetic profiles of the infected individuals [4, 17].

In terms of other proinflammatory cytokines, it is well known that IL-6 is produced by several cell lines, including antigen presenting cells (APCs) such as macrophages, dendritic cells, and B cells; IL-6 is also involved in the acute phase of inflammatory response, B cell maturation, and macrophage differentiation [21]. Patients with visceral leishmaniasis show high IL-6 serum levels during the evolution of the disease, as this cytokine acts by blocking macrophage activation, in addition to its microbicidal activity [22, 23]. It has also been suggested that IL-6 is able to control Th1/Th2 differentiation, promoting Th2 differentiation while simultaneously inhibiting Th1 polarization; it activates transcription mediated by the nuclear factor of activated T cells (NFAT), leading to the production of IL-4 by naive CD4^(+)^ T-cells and their differentiation into effector Th2 cells. IL-6 thus inhibits Th1 differentiation by upregulating suppressor of cytokine signaling- (SOCS-) 1 expression that will interfere with IFN-*γ* and the development of Th1 cells [24]. Additionally, IL-6 was recently identified as the central proinflammatory cytokine responsible for the lethal manifestations observed in pediatric patients that eventually died due to severe AVL in Piaui State, in northeastern Brazil [11].

As such, the potential pathogenic effects of IL-6 were well characterized in the present work, not only in terms of its significantly higher serum levels in the SI profile (AVL) as compared to the other profiles, but also because of the fact that it was the only cytokine that produced serum levels in the SI profile (AVL) that were significantly higher than those seen in the other profiles, confirming its strong ability to promote Th2 differentiation to the detriment of Th1 polarization, and pushing human* L. (L.) i. chagasi*-infection to the susceptible immunopathological pole of the spectrum (SI profile [AVL]). Additionally, it should be noted that even during the early onset of infection with negative DTH response (III profile) IL-6 showed significantly higher serum levels than those of TNF-*α*, IL-4, and IL-10, suggesting that it exerts a latent immunopathogenic effect even during early infection onset. It is therefore interesting to note a recent clinical finding of significantly higher IL-6 serum levels as compared to TNF-*α*, IL-4, and IL-10 in an asymptomatic 3-year-old girl with negative DTH and positive IFAT (IgG: 640) [III profile] who went on to develop classical AVL (IgG: 10.240) [SI profile] six weeks later [25].

With respect to the anti-inflammatory cytokine IL-4, a possible association between three functional IL-4 polymorphisms (−590 C/T (rs2243250), −34 C/T (rs2070874), and 70 bp VNTR [rs79071878 in intron3]) and kala-azar was recently described in an Indian cohort comprising 197 patients and 193 healthy controls—but no associations of IL-4 functional polymorphisms with the disease were identified. It was therefore postulated that other vital genes involved in the IL-4 pathway could provide genetic clues of IL-4 regulation and immunopathogenesis during kala-azar [26]. We found here that IL-4 was the anti-inflammatory cytokine showing the lowest serum levels within the entire clinical-immunological spectrum of human* L. (L.) i. chagasi*-infection, with no significant correlation (*P* ≥ 0.05) of its serum levels with any of the infection profiles (SI [AVL], SOI, III, SRI, and AI) or the control group (CG), suggesting that IL-4 has no discriminatory expression during the evolution of infection, even in regard to the pathogenic SI profile (AVL).

In terms of the other anti-inflammatory cytokines, it is well known that IL-10 is principally produced by* Leishmania*-infected macrophages, favoring the intracellular development of that parasite. Its production by CD4^(+)^ T-cells is also important for parasite maintenance, even following a clinical cure of infection, and represents a pivotal key for developing adaptive immunity against the parasite [27, 28]. It has also been observed,* in vitro*, that IL-10 is responsible for the inhibition of IFN-*γ* production, but the addition of anti-IL-10 antibodies to cell cultures converts the IFN-*γ* production and, subsequently, the lymphoproliferative responses of asymptomatic infected individuals (with positive DTH), suggesting that high levels of IL-10 are strongly associated with AVL (SI profile), making it a critical cytokine in the immunopathogenesis of this disease [12]. Interestingly, these observations are partially in agreement with the results of this study, as significantly higher IL-10 serum levels were seen in the SI profile (AVL) than in SOI or CG and in the AI and III profiles as compared to SOI, demonstrating, for the first time within the entire clinical-immunopathological spectrum of human* L. (L.) i. chagasi*-infection, that while IL-10 expression is intensively associated with AVL patients (SI profile with negative DTH), it is also associated to a lesser degree with both resistant (AI profile with positive DTH) and indeterminate individuals (III profile with negative DTH), indicating its immune-regulator role in those stages of infection. It is likewise important to emphasize our previous findings demonstrating similar IL-10 serum levels in both the AI and III profiles as compared to those in cured AVL (SI profile) patients (nine cases at least two years after treatment) [29], confirming the relevant role of this cytokine not only in the dynamics of the clinical and immunological evolution of human* L. (L.) i. chagasi*-infection [6, 7] but also in the process of AVL recovery.

When our results were examined with basis on each clinical-immunological profile of infection in relation to the proinflammatory (TNF-*α* and IL-6) and anti-inflammatory (IL-4 and IL-10) cytokines, at least four positive correlations were evident, including two between IL-6 and IL-10 in the SI (AVL) and III profiles (both marked by negative DTH responses), respectively, clearly suggesting a competition between IL-6 and IL-10 cytokines in these infection profiles evolving in the absence of a DTH response; the third was between IL-6 and TNF-*α* as seen in the III profile, which might be viewed as an attempt of the innate immune response to ensure a protective response against infection in indeterminate asymptomatic individuals (III profile). As such, it is interesting to note that follow-up efforts of 49 III cases examined in the present study failed to identify a single individual that progressed to a SI profile (AVL) after one year. Additionally, taking into account that the SRI and AI profiles (both marked by positive DTH response) represent subsequent stages of infection (III profile) among individuals with resistant genetic background, it is interesting to note that our results failed to show any difference between IL-6 and TNF-*α* serum levels in either of those two profiles, reinforcing the possibility that a positive correlation between these cytokines in the III profile might favors a protective innate immune response against infection; the fourth was between the IL-4 and TNF-*α* cytokines, also in the III profile, suggesting, again, competition between anti-inflammatory (IL-4) and proinflammatory (TNF-*α*) cytokines in infection profiles developing in the absence of a DTH response.

## 5. Conclusion

The results of the present work provide strong evidence for the association of IL-6 and IL-10 cytokines with the immunopathogenesis of the extremely pathogenic SI profile (AVL) and likewise clarify the role of proinflammatory (TNF-*α* and IL-6) and anti-inflammatory (IL-4 and IL-10) cytokines within the clinical-immunological spectrum of human* L. (L.) i. chagasi*-infection.
